# Supplementary material for: Are carbon emissions trading and green financial instruments synergistic? -Comprehensive quantitative research based on content analysis
Source: PLoS One. 2024 Mar 7;19(3):e0298601. doi: 10.1371/journal.pone.0298601 (PMC10919601; doi:10.1371/journal.pone.0298601)
Supplement: S1 Data — (ZIP) [file pone.0298601.s001.zip › Data/地级市Z_F工作报告.zip!/╔╜╬≈/╦╖╓▌╩╨/└┤╫╘╣1⁄2╓┌║┼í╛┬φ┐╦╩2╛▌═°í┐_2006.docx]

各位代表：

　　现在，我代表市人民政府，向大会作关于我市国民经济和社会发展第十一个五年规划纲要的报告，请各位代表连同《纲要（草案）》一并审议，并请各位政协委员和其他列席人员提出意见。

　　 一、“十五”时期，是我市经济社会跨越式发展的重要时期

　　过去五年，全市人民在中共朔州市委的正确领导下，坚持以邓小平理论和“三个代表”重要思想为指导，以科学发展观为统领，团结拼搏，开拓奋进，全市经济社会各个方面都取得了很大成就，成为建市以来发展最快、效益最好、人民得到实惠最多的时期之一。

　　国民经济强劲快速增长，综合经济实力显著增强。2005年，全市地区生产总值达到181.65亿元，为“九五”末的2.1倍，年均增长11.6%。财政收入保持了前所未有的高增长势头，总收入达到35亿元，为“九五”末的5.4倍，年均增长40%;一般预算收入达到11.3亿元，为“九五”末的2.8倍，年均增长22%.工业经济加速发展。增加值达到“九五”末的3.1倍，年均增长18.5%;实现利税达到“九五”末的4.8倍，年均增长37%。商贸经济繁荣活跃，社会消费品零售总额达到“九五”末的1.6倍，年均增长10.2%。

　　结构调整势头越来越好，“两大基地”建设取得明显成效。共铺开各类调产项目346个，建成投产192个。农业农村经济结构不断优化，生态率牧基地建设取得重大突破。初步形成粮、经、饲三元种植格局和优质玉米、马铃薯、小杂粮、延秋瓜菜四大生产基地;完成植树造林250万亩，种植牧草120万亩，林草覆被率达到27.5%;奶牛存栏达到13.3万头，羊160万只，分别比“九五”末增长95.6%和43.5%，畜牧业已成为农业总收入的半壁江山。新型能源和工业基地建设稳步推进。新增煤炭生产能力3000万吨，新增洗选能力5000万吨，新增电力装机容量110万千瓦，乳品加工能力由21万吨提高到53万吨，陶瓷生产能力由3亿件增加到7亿件，以硅铝、硅锰合金为主的新型冶金业和以高岭土、单晶莫来石为主的新型建材业快速兴起，以醇醚燃料为主的煤化工业开始起步，一批新的特色旅游景点建设取得了实质性进展。

　　改革开放不断深入扩大，经济发展活力不断增强。积极推进了农村税费改革，全部减免了农业税及其附加。进一步引深了国有企业改革，国企改制面达到90%.建立健全了国有资产监管体系和安全生产监督体系，完善了矿产资源保护开发和有偿使用机制。稳步推进了财政管理体制、粮食流通体制改革。切实加大了开放引进的力度，20多家国内外大企业大集团汇聚我市，兴建各类项目120多个，完成投资80多亿元。

　　基础设施建设扎实推进，发展环境不断改善。全社会固定资产投资累计达到246.7亿元，为“九五”时期的3.5倍，是建市以来投入规模最大的时期。公路建设取得重大突破。累计投资50多亿元，建成了大运高速公路朔州段、平朔一级公路、世纪大道、南环路等一批干线公路，完成了5条县际公路、10条二级公路和15条通乡油路建设任务，新增通车里程1091公里，公路密度和人均里程均居全省第一。特别是村村通油（水泥）路工程，自2003年以来，一年一个大突破，全市基本实现村村通油（水泥）路。城市建设与管理水平进一步提高。高起点进行了城市总体规划的修编，完成了安泰街、民福东街、开发北延路等一批城市具干道路和体育广场、汉墓博物馆、市二中、市三小等一批城市公共设施的建设，推进了以北关路、马邑北路为主的旧城改造和以开发区为主的新区建设，中心城市人均公共道路、人均住房面积和人均公共绿地均居全省第一。县城和小集镇建也取得了新进展。农田水利基本建进一步加强，累计完成投资6.9亿元，完成水保治理262万亩，新增节水面积31.5万亩，解决了60.8万人的饮水困难。

　　各项社会事业蒸蒸日上，精神文明和民主法制建设得到加强。建立健全了科技进一步激励机制，设立了科技进一步奖励基金。组织实施市级以上科技项目249项，科技对经济增长的贡献率进一步提高。教育事业快速发展。全面完成了“普九”任务，基本完成农村中小学危房改造，职业教育、成人教育、民办教育进一步发展，高等教育开始起步，教育质量稳步提高。卫生事业取得较大突破，疾病孩控体系和医疗救治体系建设基本完成，突发公共卫生事件应急指挥系统全面建立，新型农村合作医疗记点在怀仁、平鲁民开。文化体育活动空前活跃，承办了一系列全国、全省性文化体育活动，人民群众精神文化生活更加丰富。广播电视“村村通”工程圆满完成，覆盖全市的有线数字网络正在建设。人口和计划生育工作有了明显进步，环境达标成果得到巩固提升。思想道理建设和政治理论宣传教育深入开展，群众性文明创建活动形式更加多样。民主法制建设得到加强。市人民政府认真执行市人大及其常委会的决定和决议，自觉接受人大监督，积极听取市政协、各民主党派、各人民团体及各界群众意见，办理人大代表意见、建议和政协委员提案近千件。政府职能进一步转变，行政审批制度改革有序推进，“三务”公开不断拓展，引起全国全省关注。严打整治和综合治理工作不断深入，廉政建设扎实推进。土地、审计、物价、宗教、人防、信访、地震、气象、档案、老龄、残疾人事业等都取得了新成绩。

　　事关人民群众切身利益的问题得到较好解决，人民生活水平进一步提高。城乡居民收入较快增加。城镇居民人均可支配收入达到8787元，农民人均纯收入达到3081元，分别比“九五”末增长73.8%和38.6%.狠抓了农村扶贫攻坚和小康建设。5.1万人越过温饱线，完成移民搬迁5000户、近2万人，90.3万农业人口基本达小康。积极扩大就业，城镇新增就业岗位6.5万个，累计安置下岗失业人员4万多名。加快了社会保障体系建设，各项社保基金积累达到4．58亿元，近6000名国有企业下岗职工和1．6万名企业离退休人员按时足额领到了基本生活保障金和养老金；近5万名城市贫困人口纳入低保，7．1万名农村特困人口得到救助，7089名五保老人实行了财政转移支付。城镇住房人均建筑面积由“九五”末的20．7平方米提高到28．5平方米，农村住房人均建筑面积由“九五”末的1 9．5平方米提高到23．3平方米。

　　各位代表，过去五年的大好形势是有目共睹的。这是市委正确领导，市人大、市政协、各民主党派和人民团体鼎力支持，全市各级各部门和广大人民群众艰苦奋斗、不懈努力的结果。在此，我谨代表市人民政府，向为朔州改革开放和现代化建设作出重大贡献的全市人民致以崇高的敬意!向所有关心、支持朔州改革发展大业的同志们、朋友们，表示衷心的感谢!

　　二、“十一五”时期的指导思想和目标任务

　　今后五年，是全面建设小康社会的关键时期。我们正面临着一系列难得的发展机遇：国际国内经济持续向好，能源需求旺盛，使我市经济增长有了充分的市场支撑；全球性结构调整和产业转移步伐不断加快，有利于我们扩大开放引进，加快结构调整步伐；随着国家中部崛起战略的实施，政策优势将逐步显现；全市经济持续快速增长，社会全面进步，为我们今后的发展奠定了坚实的基础。只要我们抓住机遇，扎实苦干，就一定能够取得新的更大成就!

　　当然，我们也非常清醒地知道，目前我们还面临着许许多多的困难和问题。支柱产业单一、增长方式落后的问题仍很突出；农业综合生产能力尚未得到明显提高，农民增收渠道还很狭窄；社会事业发展相对滞后，就业压力、环境压力和安全生产压力与日俱增，构建和谐社会的任务十分繁重；在弱势群体救助上力量还十分有限，不少城乡居民生活还比较困难；社会治安状况群众还不够满意，投资环境还不够宽松，干部思想作风建设也有待进一步加强。所有这些问题和薄弱之处，无不令我们忧心忡忡，寝食难安! 我们一定要居安思危，加倍努力，不断予以克服和解决，决不辜负人民群众对政府的信任与厚望!

　　“十一五”时期全市经济社会发展的总的指导思想是：坚持以邓小平理论和“三个代表”重要思想为指导，以科学发展观统领经济社会发展全局，围绕全面建设小康社会的总目标，紧紧抓住调整经济结构、转变增长方式这条主线，继续推进新型能源工业和生态畜牧两大基地建设，在培育优势产业、推进新农村建设、加快城镇化进程、创新体制机制、扩大对外开放和实施科教兴市战略上有更大突破，努力实现综合经济实力、可持续发展能力、社会和谐水平和人民生活质量四个提高，为建设经济繁荣、功能完善、环境优美、文明开放的新朔州而奋斗。

　　根据这一指导思想，今后五年必须把握好五条原则：

　　一是坚持把科学发展观统领下的加快发展作为鲜明主题。坚持把发展作为第一要务，一切围绕发展，一切服从和服务于发展，千方百计加快发展的步伐，并在科学发展观的指引下，不断创新发展的理念与思路，不断拓展发展的空间与内涵，不断提高发展的质量与水平，努力实现全市经济社会更快更好发展。

　　二是坚持把调整经济结构、转变经济增长方式作为突出主线。以提高经济增长质量、增强综合经济实力和可持续发展能力为核心，着力构建多元支柱产业体系，不断提高产业素质，从根本上扭转我市产业结构单一、增长方式落后的局面，努力实现速度、结构、质量和效益的统一。

　　三是坚持把改革开放和科技进步作为发展的主要动力。坚定不移地推进改革，努力突破各种体制机制障碍，营造加快发展的体制机制优势；坚定不移地扩大开放引进，为全市经济社会发展注入新的活力；坚定不移地实施科教兴市和人才强市战略，不断提高自主创新能力，努力为全市经济社会发展提供强大智力支持。

　　四是坚持把全面协调发展作为重大战略任务。按照科学发展观和构建社会主义和谐社会的要求，在加快发展中，更加重视农业、农村和农民问题，努力实现城市与农村统筹协调发展；更加重视各项社会事业的发展，努力实现经济与社会的协调共进；更加重视资源节约和环境保护，努力实现人与自然的和谐相处。

　　五是坚持把不断提高人民生活水平作为发展的根本目的。坚持把人民群众利益放在第一位，把不断提高人民生活水平作为一切工作的出发点和落脚点，切实解决好事关民生民计的各类重大问题，让广大人民群众在改革发展中得到更多实惠。

　　“十一五”时期全市经济社会发展的主要目标是：

　　——经济发展再上一个新台阶。到2010年，地区生产总值达到370亿元，年均增长11％；全社会固定资产投资累计达到650亿元，年均增长20％；工业增加值达到220亿元，年均增长工7％；财政总收入和一般预算收入分别达到75亿元和22亿元，比“十五”末翻一番。

　　——经济结构更趋合理。农业建成全省一流的生态畜牧基地和特色鲜明的现代农业生产基地；工业建成全省靠前的新型能源基地，农畜产品加工、现代煤化工、新材料、陶瓷等新兴产业力争全省上游；旅游业、服务业得到大力发展，形成农业现代化、工业多元化、三产规模化的产业格局。

　　——市场经济体制更趋完善。国企改制任务全面完成，绝大部分企业建立起比较规范的现代企业制度；公共财政体制、新的投融资体制基本建立，资本、技术、人才等要素市场充分发育。非公有制经济快速发展。经济外向度明显提高，招商引资取得重大突破，外来直接投资达到300亿元。

　　——发展环境更加优越。城市功能大幅度提升，城市化水平达到47％。资源节约型和环境友好型社会建设取得明显成效，全市森林覆盖率达到23％，城市绿化率达到45％，万元地区生产总值综合能耗和平均耗水量分别下降25％和35％，力争建成国家级生态园林城市和环保模范城市。软环境得到更大力度整治，干事创业的环境更加宽松。

　　——各项社会事业更快发展。科教兴市战略得到更好实施，基础教育水平进一步提升，高等教育取得重大突破，全市高等教育毛入学率力争达到27％以上，达到或超过全省平均水平；科技在经济增长中的贡献率明显提高，新型工业化水平达到50％。文化体育事业、精神文明建设、民主法制建设等都要取得新进展。

　　——人民生活更加宽裕。城乡居民收入不断增加，城镇居民人均可支配收入达到1．48万元，年均增长11％；农民人均纯收入达到4600元，年均增长8％。就业服务体系进一步健全，社会保障水平明显提高。居住、交通、教育、卫生条件大为改善。

　　同时，我们制定了包括经济增长、社会发展、科技进步、资源环境保护和人民生活五个方面共四十四项指标组成的“十一五”经济社会发展新的指标体系。这是一个更加全面、更能体现科学发展观丰富内涵的指标体系。

　　各位代表，我们的目标是宏伟的，其跨越的幅度是历史少有的，其艰巨的程度也必将是前所未有的。但不论任务多么繁重，我们都将始终咬定目标不放松，务使宏伟蓝图变为壮丽现实!

　　为此，要切实抓好八个方面的工作：

　　(—)全力推进社会主义新农村建设，全面提高农业农村发展和农民生活水平

　　农业是国民经济的基础。我国社会主义建设的全部历史，就是一部“三农”奉献史。没有农业的巨大牺牲，就没有工业化的迅猛发展；没有农村的无私奉献，就没有城市的繁荣昌盛。现在，我们已经到了工业化的中后期阶段，我们有能力、也到了应该补偿农业、补偿农村、补偿农民的时候了。要把解决“三农”问题作为今后五年经济社会发展的第一战略任务，按照“生产发展、生活宽裕、乡风文明、村容整洁、管理民主”的要求，以产业为支撑，以农民为主体，以政府提供交通、通讯、教育、卫生等公共服务为依托，全力推进社会主义新农村建设，全面提高农业农村发展和农民生活水平。

　　大力发展现代农业，加快农村经济发展。走区域化发展道路，提升种植业效益。坚持以市场为导向，突出抓好百万亩优质高蛋白玉米、百万亩优质高淀粉马铃薯、百万亩特色优质小杂粮和百万亩瓜菜“四大基地”建设，到2010年，粮食产量保持在工4亿斤左右，油料产量达到1亿斤以上，瓜菜产量达到200万吨以上。走规模化发展道路，提升畜牧业发展水平。围绕雁门关生态畜牧经济区建设目标，突出良种繁育、防疫检疫、牧草生产、园区示范四个重点，建设优质奶源基地和肉牛肉羊生产基地，使畜牧业成为带动农民持续增收的主导产业。到2010年，奶牛存栏达到30万头、肉牛20万头、羊300万只、猪75万头，畜牧业总收入占到农业总收入的 60％以上，人均畜牧业收入占到农民人均纯收入的50％以上。走可持续发展道路，加强生态和水利建设。抓好南部山坡区120公里、西北山坡区150公里、朔万路100公里及大运高速公路、荣乌高速公路五条生态防护林带建设，加快西北山退耕还林工程区、南山自然保护封山育林区、龙首山生态综合治理区、城北防护林工程区和广武城五大生态区建设，完成工程造林200万亩，治理水土流失1200平方公里，新增有效灌溉面积10万亩，不断改善农业生产条件，提高农业综合生产能力。走产业化发展道路，壮大龙头企业。坚持把农业产业化作为新农村建设的全局性、方向性大事来抓，努力推动农副产品加工业更快发展，逐步形成独具特色的奶、肉、粮、油、菜、草产业集群。到2010年，力争建成70万吨鲜奶、70万吨乳品生产基地，形成营业收入20亿元以上的龙头企业1—2个，10亿元以上的龙头企业2—3个，农副产品加工转化率达到75％以上。

　　大力发展农村各项事业，促进农村社会全面进步。大力发展农村教育事业，从改善办学条件、加强师资队伍建设、调整学校布局等多方面入手，全面提高农村教育质量，着力解决农村学生流失和辍学问题，缓解城镇中小学压力。大力发展农村卫生事业，2008年实现农村新型合作医疗全覆盖，2010年乡村卫生医疗机构达标率达到80％以上。大力发展农村文化事业，建立布局合理的县、乡、村、户四级文化网络体系，争取用三到五年时间，实现农村电视数字化，80％的村建立村级文化阵地。全面启动“百万农民健身工程”，逐步建立农村体育活动场所。进一步完善农村社会保障体系，全面实施农村特困户救助和农村居民最低生活保障，开展农村养老保险，五保户集中供养率达到10％以上。

　　继续加大对“三农”的扶持力度，构建农村持续发展的支撑体系。构建长效的投资支撑体系。财政支农资金的增长要高于经常性财政收入增长；要整合各类支农资金，捆绑投入新农村建设；积极引导企业和民间资本投向新农村建设。构建完善的技术支撑体系。继续抓好农、林、牧等各类技术体系的建立和完善，重点抓好市农业科学研究所的筹建和百万亩农业高新技术试验示范区建设；抓好30个农业技术推广区域站，加大科技入户力度，提高农业科技水平。构建健全的社会化服务体系。建设一批新型农民专业合作社，推进农业标准化和农产品品牌化，提高农民进入市场的组织化程度和参与市场竞争的能力。

　　加强基础设施建设，改善农村基本条件和村容村貌。突出抓好以街巷硬化、村庄绿化、环境净化、路灯亮化、墙面美化和改水、改厨、改圈、改厕等“五化四改”为主要内容的村庄治理工程，着力解决农村脏乱差问题，改变农村落后面貌，改善农村人居环境。五年内至少要完成900个建制村的治理任务。加快农村公路建设，力争完成1万公里农村公路建设任务，90％的行政村通客车。加强农村饮水安全工程建设，力争60％的村庄安装自来水，所有村庄都要达到饮水安全标准。加快洁净能源推广，力争30％以上的农户用上沼气和液化气。启动“四电合一”信息工程，力争70％以上的农户通电话，5％以上的农户拥有计算机。

　　扎实推进扶贫开发，提高农民整体富裕水平。坚持把贫困农民的脱贫致富作为新农村建设的重点攻坚任务，继续实施整村推进、移民搬迁、培训转移和产业扶贫四大工程，继续组织动员社会各方面参与扶贫开发，力争到2010年基本解决全市500个贫困村、 10．5万名贫困人口的脱贫问题，使广大贫困农民跟上全面小康建设的步伐，享受到改革发展的成果!

　　(二)加快结构优化升级步伐，着力提高经济增长质量和效益

　　结构问题，是朔州经济发展中最为突出的问题。没有结构调整的快速推进，就难有增长方式的真正转变和增长质量的真正提高，难有综合经济实力和可持续发展能力的显著增强，难有真正意义上的工业化和现代化水平的提高。多年的实践反复证明，谁抓住了结构调整，谁就抓住了发展的先机。要把结构调整作为一项长期而紧迫的重大战略任务，坚持不懈地抓下去。

　　加快新型能源基地建设，加速新兴产业发展，构建多元支柱产业体系。重点抓好煤炭、电力两大传统产业和农畜产品加工、现代煤化工、新材料、陶瓷、旅游五个新兴支柱产业。要以规模化、洁净化、增值化、现代化为方向，坚持“资源整合、关小上大，能力置换、联合改造，淘汰落后、优化结构”的原则，认真组织好打击非法违法煤矿、资源整合和有偿使用、采煤方法改革“三大战役”，加快煤炭产业新型化步伐。加大煤炭资源整合力度，推动煤炭资源向大企业大集团集中，并借助大企业大集团的力量，加快现有煤矿技术改造和采煤方法改革，加快一批大型矿井和大型洗煤厂建设，力争一到两年内使全市地方煤矿由现在的205座压缩到100座左右。到 2010年，全市煤炭总产量达到1．2亿吨左右，原煤入洗率达到 90％以上，地方矿区资源回采率达到60％以上，形成全省靠前的新型煤炭工业基地。电力工业要坚持“上新上大、内外并举、煤电联营”的方针，积极推进大型坑口电厂建设，加快以低热值煤为燃料的超临界新型发电机组和煤矸石电厂建设，积极发展风能发电，力争2010年全市火电装机容量达到600万千瓦，风电装机容量达到 50万千瓦。

　　要高度重视新兴产业发展，加快资源型城市转型步伐。加快发展现代煤化工业。围绕醇醚和煤基油两条主线，大力推广应用煤制甲醇、煤液化等加工转化新技术，推动清洁燃料开发利用，加紧建设鲁能120万吨甲醇、国怀40万吨合成氨和60万吨尿素等重点工程，促进煤化工业的新跨越。大力发展新型材料业。充分利用煤矸石、粉煤灰及煤矿伴生资源，加快发展硬质合金材料、复合金属材料、铝型材料、高级耐火材料、高级涂料、新型墙体材料和特种水泥，重点抓好晋能“双10万吨”铝硅合金、利用粉煤灰提取氧化铝和高硅氧化物等项目，做大产业规模。要调整提高陶瓷业。大力发展精细加工，增加品种、提高档次，尽快由低档产品向中高档产品迈进。旅游业要按照“科学规划、市场运作、多元投入、打造品牌”的方针，围绕大运高速公路黄金旅游带，切实加快应县木塔、朔城区华夏文化大观园、怀仁金沙滩和清凉山、右玉生态体育、山阴广武城古代军事文化等一批特色景点景区的建设，打造以古代军事文化、边塞风光、北方民族融合佛教文化、现代工业文明和塞外生态文明为主题的旅游品牌，着力构建我们朔州自己的特色鲜明、景致多样、底蕴厚重、布局合理、交通便捷的旅游圈，彻底告别我市旅游业长期处于周边地区旅游业附庸地位的尴尬局面!

　　实施大企业大集团战略，做大做强优势骨干企业，打造经济发展的核心增长极。鼓励引导一批具有良好发展前景的优势骨干企业，通过加快技术改造、新上重点项目、产权多元化改组、搞好资本运作等多种途径，加快发展壮大步伐，着力培育一批技术先进、主业集中、主导产品突出、规模较大的企业集团，使其成为全市经济发展的主力舰队和核心增长极。重点抓好三大方阵建设。第一方阵企业销售收入达到100亿元以上，第二方阵达到50亿元以上，第三方阵达到10亿元以上。力争到2010年，平朔露矿销售收入达到150亿元左右，进入第一方阵；金海洋、国怀精煤公司、晋能朔州公司进入第二方阵；神头一电厂、二电厂、大唐电厂、古城集团、神华朔州公司、伊利朔州公司、蒙牛朔州公司等十家企业进入第三方阵。三大方阵企业销售收入占到全市工业销售收入的75％以上。

　　加快服务业发展，扩大产业规模，提升产业层次。以建设晋陕蒙区域性商贸物流中心为目标，推动服务业向规模化、集约化和现代化方向发展。改造提升批零贸易、餐饮接待等传统服务业，规划建设一批用现代信息网络技术装备起来的、功能完备的工农业产品批发市场，鼓励支持大型购物商城、高水平商业步行街和超级市场建设。积极推广现代流通方式，引进和建设一批较大型连锁企业。加大城市市场开拓力度，积极培育汽车、住房、通讯、旅游、文化、健身等新型消费市场，积极发展社区商业和信息咨询、商务服务、科技服务等新型服务业。大力开拓农村市场，规划建设一批覆盖全市乡村的日用消费品和农资产品连锁店，实现连锁超市进乡村。拓展现代服务业，借助大运高速和即将开工的荣乌高速互交过境的区位优势，积极发展与京津环渤海地区有效对接的大型综合物流中心，扶持发展现代仓储、联运、配送、展览展示、电子商务中心。加快资金、技术、人才等要素市场建设。

　　(三)加快城镇化进程，增强城镇综合竞争力和辐射带动力

　　城镇化是工业化和现代化的重要标志，是经济社会更快发展的必由之路，是优化城乡经济社会结构，实现城乡统筹发展、社会整体进步、人民共同富裕的内在要求和强大动力。加快城镇化进程，早已成为世界性大趋势并在继续加速发展。我们必须紧跟历史前进的步伐，大幅度提高城镇化水平，促进各种社会资源和产业、人口的整合集聚，营造经济社会发展的新平台和新优势。

　　坚持拓展规模与完善功能相结合，加快中心城市发展。加快以平朔生活区和开发区为主的新区建设，实现城市规模的快速扩张，逐步建成全市行政文化中心区；旧城区要加快改造步伐，加快第三产业发展，建成商贸旅游服务区；神头以建设现代电力工业园为主体，建成重要工业生产区；井坪要建成新型煤化工工业区，使整个市区逐步形成城北森林区、城中行政文化区、城南商贸旅游区、城东和城西北现代工业区几大板块有机结合、各有侧重的发展格局。要突出城市社会功能的完善，特别要下大决心、花大力气，着力提升城市的科技、教育、文化功能。要建设两所大学，大幅度提高教育事业发展水平；建设一座集会展、演出、文化娱乐于一体的大型文化活动中心，建设市级图书大厦和体育馆，组建一支高起点、高规格的市级歌舞团，丰富人民群众精神文化生活；建设青少年科技文化艺术活动中心，不断提高广大青少年的文化艺术修养；引进投资，建设1——2座五星级宾馆，改善我市对外形象。要不断改善城市面貌，努力提高城市品位。加快城中村改造，力争两到三年内完成小村、贺家河、北邢家河、胡家窑、北旺庄等城中村的改造任务；继续加快城北万亩森林、城中七里河水系、城南崇福寺文脉三大标志性工程建设，凸显城市特色。加快城市绿化步伐，大幅度提高城市绿化水平，努力实现“城在林中、林在城中、一半森林一半城”的生态园林城市建设目标。加强城市管理，合理划分市区两级事权，建立统一高效的城市管理体系，切实解决好城市脏乱差问题，提高城市文明程度。力争到2010年，基本建成人口接近35万人、建成区面积35平方公里、功能齐全、环境优美、生活舒适的现代化城市。

　　坚持特色化发展方向，加快小城镇建设。高起点、高标准推进县城建设，进一步加大基础设施建设力度，不断完善基本功能，改善人居创业环境，努力走出一条各具特色的发展之路。除商贸服务业外，山阴县城力求建成以乳品、化工为主的县域中心，怀仁县城力求建成以陶瓷、建材、化工、医药为主的县域中心，应县、右玉县城力求建成以农畜产品加工业和旅游业为主的县域中心。小集镇建设要立足比较优势，与产业发展、市场建设、移民并村和新农村建设相结合，大力发展城郊型、工矿型、商贸型、旅游型集镇，重点抓好神头、古城、右卫、下社、南河种、金沙滩、凤凰城等建制镇建设，带动周边农村发展。

　　抓好三大关键要素，确保城镇化建设健康扎实推进。一是加快以交通为主的各项基础设施建设。要建设西环路，与世纪大道、南环路、平朔路共同形成完整的城市外环。完成荣乌高速公路朔州段建设，实现县县通高速。加快县乡骨干公路建设和改造，高标准完成村村通油(水泥)路任务，确保市到县区、县区到乡村两个“一小时通达”。建设北同蒲铁路应县取直线，积极推动右玉至岱海铁路建设，力争县县通铁路。二是加快城镇经济发展。城镇带动力的核心是经济带动力。要把城镇经济发展作为城镇化建设的根本，高度重视市级经济发展，不断充实市级经济实力；积极发展县域经济，紧紧依托县城和集镇这一农村城镇化的前沿，合理布局生产力，努力把县城和大的集镇建设成为具有生产、贸易、交通、居住、消费等多种功能的县域经济增长中心。三是形成有利于促进农村剩余劳动力向城镇转移的体制机制。城镇化进程，就是农民进城并扎根立业的过程。要把扩大农民就业、促进农村剩余劳动力转移作为城镇经济发展的重要任务，在产业选择、区域布局上充分考虑农民就业问题。加大对进城农民自主创业的扶持。推进户籍制度改革，消除各种歧视政策，让进城农民与城市市民逐步走向平等。千方百计降低进城农民的生产生活成本，使农民既能进得来、有事干，又能留得下、住得起。

　　(四)加大改革开放力度，为经济社会发展增添新的活力

　　朔州在改革开放中诞生，还必须在改革开放中发展。要紧扣影响和制约全市经济社会发展的突出矛盾与问题，坚持以改革开放扫除各种发展障碍，拓展发展空间，增添发展动力，推动全市经济社会又快又好发展。

　　全面推进各项改革。继续推进国有企业改革，抓好已改制企业的规范完善。鼓励引导各类优势骨干企业加快产权多元化改组，积极争取上市，加快做强做大步伐。加快建立现代企业制度，建立完善法人治理结构和有利于调动各方面积极性的分配激励机制及约束机制。深化投融资体制改革。加快地方金融创新，鼓励发展风险投资、股权融资、信用担保等非银行金融机构；加快农村信用社改革；积极探索政府、银行、企业三方沟通协调机制，改善金融服务环境。加快土地使用制度创新，扩大土地市场化配置范围。深化财税体制改革。按照全省统一安排，完成县乡财政管理体制改革；加强公共财政体系建设，继续深化部门预算、国库集中收付、政府采购、非税收入收支两条线等管理制度改革。大力推进粮食流通体制改革。进一步加快住房制度改革，全面启动县区住房公积金制度。

　　放手发展民营经济。要把加快民营经济发展作为激发整体经济活力和建立社会主义市场经济体系的重要突破口，作为改变我市产业结构单一畸重局面的有力杠杆，进一步放宽市场准入，放宽行政审批，努力拓宽民营经济发展的空间。要建立有效的帮扶体系。建立中小企业融资风险基金，搭建中小企业从政策性银行融资的平台。建立中小企业发展基金，扶持发展民营高科技、资源深加工和农畜产品加工等项目。加快建立信息服务、创业辅导、人才培育、法律援助、行业协会等服务体系，激发民间投资创业热情。支持、鼓励民营企业参与国有经济的改组改制，培育一批带动性强的骨干企业。疏通优秀人才进入民营企业的渠道，通过有效的保障机制，引导机关人员到民营企业工作；鼓励吸引大中专毕业生及市内外各类人才在我市创办民营企业或到民营企业工作。指导督促民营中小企业加强诚信建设，提高信用融资能力；打造优质品牌，提高市场竞争力；建立现代管理制度，推动家族企业逐步向产权社会化转变。

　　全力扩大开放引进。唯有大开放，才有大发展；谁走在对外开放的前列，谁就能占领发展的制高点。要牢固树立“以大开放促进大引进、以大引进促进大发展”的思想，坚持把扩大开放引进作为保障经济投入、加快结构调整步伐、推动全市经济社会更快更好发展的关键环节和主要依托力量，举全市之力，努力实现开放引进的大突破、大跨越。要进一步解放思想，坚决破除封闭自卑、无所作为的心态，超越自然环境所造成的地理和心理局限，增强开放引进的信心和决心；坚决摒弃固步自封、怕担责任的保守思想，大力弘扬敢为人先、敢冒风险、勇于探索、勇于创新的精神。进一步加强组织领导，建立开放引进目标责任体系和考核评价体系，把开放引进作为考核干部政绩的突出内容；建立和完善招商引资协调机制，及时解决招商引资工作中的新矛盾、新问题；认真抓好各类优惠政策的制定、完善和兑现，调动全社会力量参与招商引资。坚决破除“肥水不流外人田”的狭隘意识，敢于以优势企业和优质资产来吸引和利用外来资金。不断拓宽开放领域，凡是国家没有明令禁止的行业和领域，都要全面放开；凡是有碍于开放引进的条条框框，都要坚决清理废除。不断丰富开放引进的内涵，既注重经济往来，也注重科技、教育、文化的交流合作。要采取灵活多样的方式，坚持“走出去”与“请进来”相结合，认真组织好各种招商引资活动，推出精品项目，提高招商引资的成功率。加大软环境治理力度，努力营造亲商、安商、扶商、富商的氛围，增强对外吸引力。要打好资源牌，以资源换资金，以资源换项目，以资源换发展，适当提高能源工业准入门槛，凡投资煤炭采掘业者，都要求其配套建设一个煤炭深加工或其他非煤项目，走出一条以地下促地上、资源型城市可持续发展的新路子。

　　(五)大力实施科教兴市和人才强市战略，积极发展各项社会事业

　　进一步加快教育事业发展。巩固提升九年制义务教育，力争到 2008年全市义务教育全面达标。努力拓展优质高中教育，在校生人数要由现在的3．1万人扩大到6万人左右，60％以上的高中学校达到优质教育标准。强化职业技术教育和成人教育，市县两级力争各办好2所中等职业技术学校或职业高中，加快适用人才培养。进一步加大对教育的投入和扶持力度，保证财政投入持续稳定增长，鼓励社会各方兴办民办学校。从今年起，用两年时间全部免除农村和城市特困家庭义务教育阶段学生学杂费，继续对农村义务教育阶段贫困家庭学生免费提供教科书并补助寄宿生生活费。不断提高科技发展水平。把依靠科技进步、增强自主创新能力作为调整经济结构、转变经济增长方式的主要动力，加快地区创新体系和科技中介服务体系建设，加快技术引进、消化、吸收和创新，务求在促进全市产业结构优化升级、高新技术产业发展、推进循环经济与资源节约、提高农业综合生产能力等方面的共性技术和关键技术的研究推广上取得重大突破。完善科技创业投资机制，加大科研资金投入，设立高新技术产业发展基金及高新技术投资风险基金，加快培育和发展科技型民营企业，大力推进科技园区建设。认真实施人才强市战略，紧紧抓住培养、吸引、使用三个环节，不断完善选人用人、利益激励、人才流动三大机制，努力营造人才辈出、人尽其才的社会氛围。大力发展卫生事业。进一步强化疾病防控体系和医疗救治体系建设，加强基层医疗卫生设施建设，大幅度改善全市医疗卫生条件；不断深化医疗卫生体制改革，加强医疗卫生市场整顿，切实解决好广大群众看病难、看病贵和因病致贫、因病返贫问题。加强城乡文体设施建设和广播电视网络建设，开展多种形式的文化体育活动，丰富人民群众精神生活。强化人口和计划生育I作，稳定低生育水平。继续发展档案、史志、气象、测绘、防震减灾等事业，关心支持老龄、妇女、儿童、残疾人、民族宗教等工作。加强国防教育和国防后备力量建设，切实抓好民兵预备役“双应一体化”和“双服务”工作的落实。加强社会主义精神文明和公民思想道德建设，扎实推进“三个代表”重要思想、科学发展观和“八荣八耻”教育，深入开展文明单位、文明村镇、文明社区等群众性创建活动。

　　(六)加强资源环境保护，建设资源节约型和环境友好型社会

　　坚持节约与开发并重、节约优先的方针，以提高资源利用率为核心，着力构建节约型产业结构、生产体系和消费方式。大力推进能源节约，抓好冶金、电力、化工、建材等重点耗能行业和企业的节约工作，积极推进热电联产、余热利用、建筑节能、政府机构节能、绿色照明等节能工程；大力发展节水型农业，积极推进工矿企业水循环利用，加快煤、电、冶等高耗水行业工艺设备和技术流程改造，完善城市节水设施，提高废水、中水回收利用；严格耕地保护。大力发展循环经济，认真抓好煤电冶、煤矸石发电和粉煤灰综合利用、煤层气和煤化工开发等产业链条，促进资源综合利用。全面推进清洁生产，从源头上减少废料产生。编制并实施建设资源节约型社会行动纲要和考核评价办法，通过法规政策和价格杠杆，引导激发全社会节能降耗的积极性和主动性。

　　大力实施“蓝天碧水”工程，全方位推进环境整治。进一步强化各级政府和企业防治环境污染目标责任制；严格实行污染物排放总量控制和排污许可制度；严格实行分类控制，城市周边一定范围杜绝再上污染严重项目，并限期改造或逐步关闭、搬迁已有污染严重企业，坚决清除城市燃煤锅炉。加强环保设施建设，所有电力、化王、建材等排污严重企业都必须安装脱硫除尘设备。加快污水、垃圾处理设施建设，扩大集中供热和清洁燃气覆盖面，积极发展环保产业，加强农村污染治理，力争使全市环境质量有明显好转。

　　(七)切实解决好群众的切身利益问题，提高和谐社会建设水平

　　坚持以人为本，执政为民，时刻把人民群众的冷暖记心头，从最基层人民群众的安危健康想起，从满足人民群众最基本的衣食温饱需求做起，自觉运用人民赋予的权力，积极为人民群众办实事、办好事，帮民富、解民忧、保民安、化民怨，促进社会公平，维护社会正义，构建和谐社会。

　　千方百计扩大就业。继续完善公共就业服务体系，努力拓宽就业渠道，积极开发就业岗位，鼓励自主创业和自谋职业。认真落实财政投入、小额贷款、免费培训，和税费减免等优惠政策，完善对困难群众的就业援助制度，建立促进扩大就业的长效机制。力争每年新增城镇就业岗位1．5万个。

　　完善社会保障体系。五年内使养老保险基本覆盖城镇各类企业职工、个体工商户和灵活就业人员。扩大失业保险范围，推动事业单位和非公有制企业参加失业保险。扩大基本医疗保险和工伤保险覆盖范围，完善城镇职工基本医疗和生育保险制度。进一步完善和落实城市低保、农村特困群众救助、大病救助、贫困家庭子女就学资助、五保户供养等十二项社会救助制度，保障困难群众最基本的生活需求。

　　进一步完善收入分配制度。坚持和完善效率优先、兼顾公平、各种生产要素按贡献参与分配的分配制度。着力提高城乡低收入者的收入水平，扩大中等收入者的比例，限制少数垄断行业的不合理收入。按照全国统一部署，改革公务员工资制度，在清理规范津贴补贴的基础上，适当提高公务员工资水平，建立职务与级别相结合的工资制度和工资正常增长机制。统筹解决企业离退休人员、城市“低保”对象、残疾军人等各类优抚对象的待遇，提高补助标准。推进事业单位收入分配制度改革。

　　切实维护社会稳定。完善安全生产管理体制和机制，增加安全生产投入、消除各种隐患。特别要加大煤矿安全生产专项整治和执法监察力度，坚决杜绝重特大事故发生。加强市场秩序整顿，确保食品、药品安全。重视处理好农村征地、城镇房屋拆迁、国有企业改制等涉及群众利益的问题。加强信访工作，依法、及时、合理地处理好群众反映的问题。推进社会治安综合治理，严厉打击各种刑事犯罪，维护正常社会秩序，确保人民群众生命财产安全。

　　(八)加强政府自身建设，营造良好经济社会发展环境

　　致力科学发展、促进社会和谐，对政府工作提出了新的要求。必须切实加强政府自身改革和建设，着力提高政府公信力、凝聚力和战斗力。

　　加快建设服务型政府。进一步转变政府职能，改善管理经济的方式，加强社会管理和公共服务，突出抓好各类基础设施及科技、教育、文化、卫生等公共产品的提供。加快由“全能政府”向“有限政府”的转变，坚决把政府不该管的事情交给市场、企业和社会中介机构，使市场经济能够更加自由地发展。坚持不与民争利，政府退出一切竞争性领域，使市场经济拥有更力口充分的发展空间。大力促进民营经济、中小企业等市场主体的发育，使其如雨后春笋般成长于朔州大地。

　　加快建设务实高效政府。坚持求真务实，讲实话、办实事、求实效，力戒空谈和浮夸，克服形式主义和官僚主义。进一步引深行政审批和行政许可制度改革，严格执行首办负责制和限时办结制，提高办事效率和服务水平。坚持从严治政，防止政府权力部门化、部门权力个人化，坚决惩治行政不作为和乱作为，确保政令畅通，杜绝推诿扯皮。

　　加快建设诚信法治政府。各级政府要言必行、行必果，凡是对社会和人民作出的承诺，都要务必兑现；要保持政策的连续性、稳定性、长久性，增强经济发展的可预见性，以诚信政府的建设带动信用社会的建设。要带头遵纪守法，严格依法行政，积极推进“五五”普法教育，力口快建立权责明确、行为规范、监督有效、保障有力的行政执法体制，推行行政执法责任制，促进文明执法、公正执法。建立健全有效的行政监督体制和机制。各级政府要自觉接受同级人大及其常委会的监督，认真执行人大决议，定期向人大报告工作；积极接受人民政协的民主监督；认真听取各民主党派、无党派人士和各人民团体的意见；加强基层民主建设，继续推进“三务”公开，强化基层民主监督；拓宽新闻监督、公众监督渠道，做到有权必有责、用权受监督、违法受追究、侵权要赔偿。

　　加快建设勤政廉洁政府。全面落实党风廉政建设责任制，建立健全教育、制度、监督并重的惩治和预防腐败体系，严格执行领导干部经济责任审计和重点资金、重点项目、重点工程审计，健全土地出让、建设项目招投标、资产交易和政府采购制度，保持政府各部门和全体工作人员廉洁办事的良好风气。

　　实现“十一五”宏伟目标，是一项光荣而艰巨的历史性任务。各级政府工作人员，特别是各级领导干部，要忠于职守，勤勉尽责，夙兴夜寐，只争朝夕，以高度的责任感、使命感和加倍的工作热情、创业激情去迎接这一挑战，为建设新朔州贡献智慧和力量。

　　三、团结拼搏，奋发进取，确保“十一五”良好开局

　　今年是“十一五”规划的开局之年、起步之年。做好今年的工作，意义重大而深远。我们一定要格外珍惜目前的大好形势，以更加振奋的精神，更加昂扬的斗志，更加务实的作风，团结拼搏，乘胜前进，坚决打好“十一五”经济社会发展第一仗，确保“十一五”规划有一个良好开端。全市地区生产总值要达到215亿元，增长11％；财政总收入要达到43．68亿元，增长24．6％；城镇居民人均可支配收入和农民人均纯收入分别达到9840元和3330元，增长12％和8％。农业农村要更快发展。优质高蛋白玉米、优质高淀粉马铃薯、优质小杂粮和瓜菜面积分别达到150万亩、100万亩、100万亩和60万亩，粮食总产达到14亿斤，蔬菜总产达到100万吨；奶牛存栏达到14．5万头；完成植树造林49．8万亩；完成整村推进脱贫12个；100个新农村试点全面铺开。结构调整要迈出新步伐。集中抓好33个重点工业调产项目，其中续建9个，力争新开工14个，前期推进10个。扎实推进煤炭资源整合和采煤方法改革，坚决关闭9万吨以下煤矿，30万吨以上煤矿全部实行采煤方法改革，切实抓好20座百万吨以上大型矿井建设。基础设施建设要再掀新高潮。公路建设要完成元元公路、虎山线右玉至杀虎口段、应县南环路、右玉通市油路四条县乡公路的改造建设，争取荣乌高速公路朔州段、西环路和大忻线怀仁至阳方口段建设改造工程开工；城市建设重点抓好大运公路市区段改造等工程。各项社会事业发展要取得新成效。农村中小学布局调整、教育人事制度改革要取得较大突破；市中心医院、疾控中心、急救中心建设要全面完成。文化、体育、计划生育、社会保障体系建设等都要取得新进展。

　　同时，市人民政府要在市委的正确领导和各级政府的支持配合下，切实为全市人民办一些好事、实事。其中，落实省政府的实事十二件：一是建设安康住房2900套30万平方米；二是改造工矿企业棚户区5万平方米；三是培训农民5万人，转移农村剩余劳动力 3万人；四是在全市推行医疗救助制度；五是解决104个村10万人的饮水安全；六是解决6300名山区贫困人口的温饱问题，完成扶贫移民1300人，保障6．09万名特困群众的基本生活，解决1782名五保老人的供养问题；七是提高各类优抚对象生活补助标准；八是新建改建城镇中小学标准化操场53个；九是继续对农村贫困中小学学生实行“两免一补”；十是推进城市集中供热和大气治理工程；十一是完成通达工程、村村通油(水泥)路和农村街道硬化工程1500公里，新建乡镇汽车站6个；十二是发展农村连锁店 68个。在此基础上，市政府还要为群众办好九件实事：一是新建、改建100所农村寄宿制学校；二是开工建设市职业技术学院；三是改扩建60个乡镇卫生院和100个村级医疗服务所；四是开工建设七里河治理工程；五是启动小村、贺家河、北邢家河三个“城中村”改造；六是建设综合文化活动中心；七是在中心市区兴建20个公厕；八是建设开发北路学苑小区过街天桥；九是完成朔神大道改造工程。

　　各位代表，现在，我们已经站到了一个新的历史起点上，全市 150万人民对我们寄予厚望。我们一定不负重托，不辱使命，在中共朔州市委的坚强领导下，高举邓小平理论伟大旗帜，坚持以“三个代表”重要思想和科学发展观为指导，讲团结，树正气，抓落实，紧紧依靠全市人民的智慧和力量，开拓奋进，迎难而上，为实现“十一五”规划的宏伟目标而努力奋斗!
